# Recurrent Herpes Zoster in an Immunocompetent Male: A Case Report

**DOI:** 10.31729/jnma.6629

**Published:** 2021-11-30

**Authors:** Niraj Parajuli, Rushma Shrestha, Laila Lama, Anupama Karki

**Affiliations:** 1 Department of Dermatology and Venereology, National Academy of Medical Sciences, Bir Hospital, Mahaboudha, Kathmandu, Nepal

**Keywords:** *herpes zoster*, *immunocompetent*, *recurrent*, *shingles*, *varicella zoster*

## Abstract

Herpes zoster is an infection caused by reactivation of varicella-zoster virus presenting as multiple grouped vesicular eruptions in a dermatomal pattern with associated pain. Recurrent herpes zoster is an uncommon event in an immunocompetent host. Here, we report a case of a young male presenting with herpes zoster over the T9 and T10 dermatome with the previous scarring of herpes zoster over the T6 dermatome over the right upper trunk. The patient improved on treatment with oral acyclovir and analgesics. In any patient with recurrenrt hepes zoster, work-up should be done to rule out immunosuppresion.

## INTRODUCTION

Herpes zoster (HZ) is caused by reactivation of latent varicella zoster virus in cranial nerve or dorsal root ganglion with the spread of the virus along the sensory nerve to the dermatome presenting as vesicular eruption mostly localized to a particular dermatome. HZ is considered to be a single life event and recurrence was considered more common in immunocompromised but recent studies have shown an incidence rate of up to 6%.^1,2^ Here, we present a case of a young immunocompetent presenting with recurrent HZ.

## CASE REPORT

**Figure 1 A.**
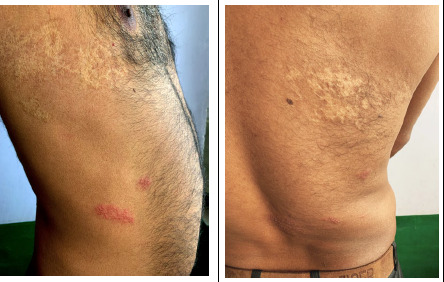
Figure showing grouped vesicles with surrounding erythema over the right flank T9, 10 dermatomes. Hypopigmented atrophic scar over over the T6 dermatome. **B.** Multiple grouped vesicles with erythema noted over the lower back arranged dermatomal pattern not crossing the midline with large hypopigmented atrophic scar over the right infrascapular region.

A 35-year-old hospital employee came to dermatology out-patient with burning sensation and pain over the right flank along with multiple skin lesions of 3 days duration. The patient also complained of mild fever and myalgia. Two days after, the patient noted multiple vesicular skin eruptions localized to the area of pain (Figure 1 A and B).

The patient had a history of HZ over the right upper trunk 5 years back, prominently visible with scarring over the dermatomal area (T6). No other significant medical or past history was noted.

Systemic examination was grossly unremarkable. On skin examination, multiple group vesicles with surrounding erythema were noted over the right flank following a dermatomal pattern. A blood profile was done which was negative for human immunodeficiency virus (HIV), hepatitis B surface antigen (HBsAg), and Hepatitis C virus. A complete blood report was within normal limits and random blood sugar was 150mg/dl. Polymerase chain reaction for VZV and Herpes simples was not done due to unavailability in our hospital.

From the clinical features of multiple grouped vesicles in a dermatomal pattern, the previous scarring over a dermatome, we diagnosed it as a case of recurrent herpes zoster. The patient was started on Oral Acyclovir 800 mg five times a day for 1 week and tab Acetaminophen 500 mg thrice daily. All lesions healed with symptomatic improvement noted in a week.

## DISCUSSION

Recurrent HZ is defined as the appearance of HZ in a patient with a previously infected person with a dermatomal rash and pain.^3^ The diagnosis is made clinically in most cases however, the polymerase chain reaction can confirm atypical cases.^4^ Treatment of HZ includes optimal and early use of antiviral therapy and analgesics. Similarly, a new recombinant zoster vaccine is useful in reducing the incidence of HZ by more than 90%. It had been postulated that risk of recurrence is associated with increasing age, immunosuppression, emotional or physical stress, fever, and exposure to ultraviolet light.^5^ HZ reactivation has also been reported following trivalent influenza, hepatitis A and rabies vaccine suggesting vaccine-modulated immunomodulation.^5^ Post-herpetic neuralgia during the first episode of HZ is significantly associated with recurrence.^2^ Recurrent HZ is an uncommon event with a global recurrence rate found varying from as low as 1% to up to 6%.^2,3,6^ Treatment for recurrent HZ is similar to that of first episode with antivirals and supportive medications including analgesics.

No reports of recurrent herpes zoster has been published from Nepal till date. The case is presented here due to the rarity of such incidents in an immunocompetent host.

Recurrent HZ is not an uncommon event and can occur even in immunocompetent host. But a thorough investigation should be done to find out any systemic illness to rule out any cause for immunosuppression.
